# 

**Published:** 1786

**Authors:** 


### CATALOGUE OF BOOKS.

1. THE Edinburgh New Diſpenſatory: containing, 1. Elements of pharmaceutical chemiſtry; 2. The materia medica, or an account of the ſubſtances employed in medicine, with the virtues and uſes of each article, ſo far as they are warranted by experience and obſervation; 3. Pharmaceutical preparations; 4. Medicinal compoſitions; the two latter parts comprehending the preparations and compoſitions of the laſt London and Edinburgh Pharmacopeias, with ſuch old ones as are kept in the ſhops; the whole interſperſed with practical cautions and obſervations; being an attempt to collect and apply the later diſcoveries to the Diſpenſatory publiſhed by William Lewis, M. B. F. R. S.; with new tables of elective attractions, ſingle and double, of antimony, mercury, mercury, &c. and copper-plates of pharmaceutical inſtruments. By *Charles Webſter*, M. D. and *Ralph Irving*, M. D. 8vo. Edinburgh, 1785.

2. Medical Commentaries for the year 1785; exhibiting a conciſe view of the lateſt and moſt important diſcoveries in medicine and medical philoſophy. Collected and publiſhed by *Andrew Duncan*, M. D. F. R. and A. S. Ed. Phyſician to His Royal Highneſs the Prince of Wales for Scotland, Fellow of the Royal College of Phyſicians, Edinburgh, and Member of the Royal Societies of Medicine of Paris, Copenhagen, Edinburgh, &c. Volume the Tenth. 8vo. *Murray*, London, 1786.

3. Medical Cautions for the Conſideration of Invalids, more eſpecially of thoſe who reſort to Bath. By *James Makittrick Adair*, M.D. Member of the Royal Medical Society, and Fellow of the College of Phyſicians, Edinburgh. Publiſhed for the benefit of the General Hoſpital at Bath. 8vo. *Dodſley*, London, 1786.

4. Experiments and Obſervations on Quilled and Red Peruvian Bark: among which are included ſome remarkable effects ariſing from the action of common bark and magneſia upon each other, other. With remarks on the nature and mode of treatment of fevers, putrid fore throat, rheumatifm, fcrophula, and other difeafes; in order to afcertain the cafes in which bark may be adminiftered, either alone, or combined with other remedies, to the beft advantage. To which is added an appendix on the Cinchona Caribbæa. By *Thomas Skeete*, M. D. 8vo. *Murray*, London, 1786.

5. The prefent Practice of Surgery: containing the defcription, caufes, and treatment of each complaint; together with the moft approved methods of operating. By *Robert White*, M. D. and Practitioner in Surgery. 8vo. *Johnfon*, London, 1786.

6. An experimental Inquiry into the Properties of Opium, and its effects on living Subjects: with obfervations on its hiftory, preparations, and ufes. Being the difputation which gained the Harveian prize for the year 1785. By *John Leigh*, M. D. 8vo. *Robinfon*, London, 1786.

7. A Letter to a Phyfician in the Country on Animal Magnetifm; with his Anfwer. 8vo. *Debrett*, London, 1786.

8. Experiments and Obfervations relating to acetous Acid, fixable Air, denfe inflammable ble Air, Oils, and Fuel; the matter of Fire and Light, metallic Reduction, Combuſtion, Fermentation, Putrefaction, Reſpiration, and other ſubjects of chemical Philoſophy. By *Bryan Higgins*, M. D. 8vo. *Cadell*, London, 1786.

9. An Inquiry into the Origin and Antiquity of the Lues Venerea; with obſervations on its introduction and progreſs in the iſlands of the South Seas. To which is added a ſhort view of the various remedies recommended in that diſtemper from its firſt appearance in Europe to theſe times; with general remarks on the preſent received modes of treatment. By *William Turnbull*, Surgeon of His Majeſty's Navy. 8vo. *Murray*, London, 1786.

10. A Narrative of the Death of Captain James Cook; to which are added ſome particulars concerning his life and character. With obſervations reſpecting the introduction of the Venereal Diſeaſe into the Sandwich Iſlands. By *David Samwell*, Surgeon of the Diſcovery. 4to. *Robinſon*, London, 1786.

The circumſtances, with reſpect to the venereal diſeaſe, mentioned in this pamphlet, ſeem to render it highly probable that the natives of the Sandwich Iſlands were afflicted with the venereal venereal diseafe before thofe iflands were difcovered by Captain Cook.

11. Differtatio Medica Inauguralis de Dyfenteria. Auctore *Nicolao Archer*, Hiberno. 8vo. Edin. 1785.

12. Differtatio Medica Inauguralis de Catarrho. Auctore *Joanne Barrow*, Anglo. 8vo. Edin. 1785.

13. Differtatio Medica Inauguralis de Apoplexia Sanguinea et Serofa. Auctore *Joanne Franklyn Clark*, Britanno. 8vo. Edin. 1785.

14. Differtatio Medica Inauguralis de Catarrho Epidemico qui anno 1782 graffabatur. Auctore *Joanne Duncan*, Scoto. 8vo. Edin. 1785.

15. Differtatio Medica Inauguralis de Pertuffi. Auctore *Edvardo Fairclough*, Hiberno. 8vo. Edin. 1785.

16. Tentamen medicum Inaugurale quædam de Emeticis complectens. Auctore *Radulpho Irving*, Scoto. 8vo. Edin. 1785.

17. Differtatio Medica Inauguralis de Cholera. Auctore *Jacobo Lyons*, Virginienfe. 8vo. Edin. 1785.

18. Differtatio Medica Inauguralis de Hepatis Inflammatione. Auctore *Samuele Macay*, Hiberno. 8vo. Edin. 1785.

19. Dissertatio Medica Inauguralis quædam de Diabete complectens. Auctore *Antonio Mann*, Hiberno. 8vo. Edin. 1785.

20. Dissertatio Medica Inauguralis de Ascite. Auctore *Henrico Moorhouse*, Anglo. 8vo. Edin. 1785.

21. Tentamen Inaugurale de Rachitide. Auctore *Cornelio Pyne*, Hiberno. 8vo. Edin. 1785.

22. Dissertatio Medica Inauguralis de Dysenteria. Auctore *Joanne R. B. Rodgers*, Americano, ex republica Novi-Eboraci. 8vo. Edin. 1785.

23. Dissertatio Medica Inauguralis de Rheumatismo acuto. Auctore *Roberto Sime*, Scoto. 8vo. Edin. 1785.

24. Dissertatio Medica Inauguralis de Ascite abdominali. Auctore *Gulielmo Spooner*, Americano. 8vo. Edin. 1785.

25. Tentamen Inaugurale quædam de Sanguinis motu et quantitate complectens. Auctore *Carolo Throckmorton*, Anglo. 8vo. Edin. 1785.

26. Tentamen Physiologicum Inaugurale de Alimentorum concoctione. Auctore *Joanne Usher*, Hiberno. 8vo. Edin. 1785.

27. Dis-

27. Diſſertatio Medica Inauguralis de Halitu Cuticulari. Auctore *Joanne Yule*, Britanno. 8vo. Edin. 1785.

28. Diſſertatio Medica Inauguralis quædam de Cantharidum Natura et Uſu complectens. Auctore *Gulielmo Puſey Hayle*, Jamaicenſi. 8vo. Lugdun. Batav. 1786.

29. Diſputatio Phyſiologica Inauguralis quædam de Generatione complectens. Auctore *Jacobo Edvardo Smith*, Anglo. 8vo. Lugdun. Batav. 1786.

30. Tentamen Chemicum Inaugurale de Connubio Chemico communi mutationum chemicarum cauſa. Auctore *Gualtero Vaughan*, Anglo. 4to. Lugd. Batav. 1786.

31. Collectanea Medica Inauguralia, ſive ſyſtematis Vaſorum abſorbentium ſuccincta Deſcriptio. Auctore *Lamberto Luca Van Meurs*, Arnhemia-Gelro. 4to. Hardevici, 1786.

32. Waernemingen op het genezen van de Gonorrhœa, en zommige andere uytwerkingen van het venerien venyn. De tweede uytgaeve door *Samuel Foart Simmons*, M. D. F. R. S. Lit van het Collegie der Genees-Heeren te London; en van de Koninglyke Genees-kundige Societeyt te Parys. Overgeſtelt uyt het Engelſch, ende vermeerdert met nuttige aenmerkingen door *F. A. Van Zandycke*, M. D. 8vo. Bruges, 1785.

33. *Ignatii Wltczek*, S. M. Regis Poloniæ Medici et Confiliarii, Philofophiæ et Medicinæ Doctoris, artis obftetriciæ magiftri, Cafus peculiaris Hiftoria. 8vo. Vilnæ, 1783.

This is the cafe of a young lady, twenty-three years old, who, after fwallowing feveral keys, (one of which meafured about three inches in length, and was with difficulty forced down by means of a probang) nails, needles, different pieces of gold, filver, and copper coin, glafs, &c., lingered twenty-two weeks; during the whole of which time fhe was afflicted with an inceffant naufea, and with frequent and painful vomiting of a fœtid, blackifh fluid. Her fæces were in general hard, and of a black colour.—On diffection, the ftomach was found in a very difeafed ftate, its inner furface being every where inflamed and ulcerated. It contained, befides the articles already enumerated, two penknives, the blades of which were feparated from the handles, the handles of fix table fpoons, and the bowls of three others, which had been bent by the patient to enable them to pafs into the ftomach.

34. Stirpes

34. Stirpes Cryptogamicæ novæ aut dubiæ, iconibus adumbratæ, additaque historia analytica illustratæ, a *Joanne Hedwig*, M. D. Folio. Lipsiæ. Fascic. I. II. 1785—6.

A number of this elegant work, containing ten plates, is to appear every six months till the whole is completed. The price to subscribers, for coloured impressions, is twelve shillings sterling for each number, and to non-subscribers sixteen shillings.

35. *Andreæ Bonn* Tabulæ Ossium morbosorum precipue Thesauri Hoviani. Fascic. I. c. Tab. vii. Folio. Amstelodam. 1785.

These plates are well engraved. The bones are represented of their natural size.

36. Delle Parotidi nei mali acuti; Dissertazione Epistolare del Sig. Dottore *Annibale Mariotti*, pubblico professore di medicina teorica nell' Universita di Perugia; socio dell' imperiale Accademia Leopoldina di Germania, &c. diretta all Sig. Ab. *Gaetano Bellini*. 8vo. Perugia, 1785.

37. Flora Pedemontana sive Enumeratio methodica Stirpium indigenarum Pedemontii. Auctore *Carolo Allionio*, in Arch. Taur. Prof. Bot. em. Hort. publ. et mus. Rer. Nat. Directore rectore prim. &c. Folio. Auguſtæ Taurinorum, 1785. Tom. III.

38. El diſcipulo inſtruido en el arte de Partear; obra muy util para los que ſe dedican a tan ſaludable Exercicio. Diſpuerto en dialogo para ſu mayor inteligencia por D. *Pedro Vidart*, Cirujano en eſta Corte, &c. 8vo. Madrid, 1785.

39. Compendio inſtructivo ſobre el mejor metodo de curar las tercianas y quartanas, con arreglo a la doctrina de Hypocrates, experimentada por el celebre Alexandro Traliano. Su Autor D. *Santiago Puig*, Medico de la S. R. H. del Refugio y del Hoſpital de la Latina, &c. 8vo. Madrid, 1786.

40. Relacion de las epidemias de calenturas putridas y malignas que en eſtos ultimos anos ſe han padecido en el Principado de Cataluna, y principalmente de la que ſe deſcubrio el ano paſado de 1783 en la Cuidad de Lerida. Por D. *Joſeph Maſdevall*, Medico del Rey nueſtro Senor con exercicio, &c. 4to. Madrid, 1786.

41. Queſtio Medica, eaque Therapeutica, an dolori Colico Opium? quam Præſide *D. J. A. Tournay*, pro Doctoratu medico propugnavit *Joannes Nicolaus Lamoureux*, ex Albercheville, Dioecefis Metenſis. 4to, Nanceii, 1784.

42. Differ-

42. Diſſertatio Pathologica Inauguralis de Reſorbtione læſa, ut morborum cauſa. Auctore *Nicolao Damaſo Marchand*, Petro-Pontino. 4to. Nanceii, 1785.

43 Diſſertatio Medica Practica de Ictero. Auctore *Claudio Anna Franciſco Petro Pichon*, Abrincino. 4to. Nanceii, 1785.

44. Diſſertatio Medica Inauguralis de Vomitu Cruento. Auctore *Bernardo Grillot*, Plumberiano. 4to. Nanceii, 1785.

45. Diſſertatio Anatomico-Phyſiologica de Nervis. Auctore *Petro Ludovico Urbano Coſte*, Geſienſi. 4to. Nanceii, 1786.

46. Diſſertatio Inauguralis Medica de Vi vitali Arteriarum. Auctore *Chriſtiano Kramp*, Argentienſi. 8vo. Argentorati, 1785.

47. Elogio Hiſtorico de Don *Joſeph Quer*, Cirujano Conſultor del Exercito, Academico del Inſtituto de Bolonia, de la Real Academia Medica Matritenſe, y primer Profeſſor del Real Jardin Botanico de Madrid: le eſcribia el Dr. Don *Caſimiro Gomez de Ortega*, primer Catedratico del miſmo real Jardin Botanico, Juez examinador perpetuo de Pharmacia, Secretario de la Real Academia Medica de Madrid para las Correſpondencias extrangeras, individuo de la de las ciencias de Paris, de la Real Sociedad de

Londres,

Londres, del Instituto de Bolonia, y del numero de la Real de la Historia. 4to. Madrid, 1784.

48. Memoire et Observations sur les Maladies des ouvriers de la Digue et des habitans du nouveau Poldre d'Arenberg, avec la maniere simple et facile employée pour les guerir, conformement au voeu de la Nature, et au principe si recommendé par Hypocrate, *quo natura vergit*, &c. et ailleurs, *nihil fingendum, aut excogitandum, nisi quod natura ferat aut faciat.* Par M. *le Roux*, D. M. Ancien Chirurgien d'Etat des Armées Imperiales et Royales. 8vo. Bruxelles, 1785.

49. Tentamen ΨΥΧΟ-ΣΩΜΑΤΟ-ΙΑΤΡΙΚΟΝ, seu Conspectus Thesiformis, de Natura Animæ et Corporis, sive de Spiritu et materia quatenus medicinam spectant. Auctore *Marco Lemort Demetigny*, Gandavensi. 4to. Monspelii, 1784.

50. De Scilla, Dissertatio Inauguralis Medica. Auctore *Philippo Henrico Caspari*, Schauemburgensi. 4to. Goettingæ, 1785.

51. Analecta de Antimonii crudi et Antimonialium præcipuorum usu medico. Dissertatio Inauguralis Auctore *Raph. Hermanno Stender*, Curono. 4to. Gottingæ, 1785.

